# Account of an Epidemic Fever Which Occurred at North Boston, Erie County N. Y. during the Months of October and November 1843

**Published:** 1845-09-01

**Authors:** Austin Flint

**Affiliations:** Buffalo N. Y.


☞ The following article has been handed us for publication by a friend and correspondent, who will be recognised by his subscribed initials, as the author of two interesting communications in the first and second numbers of the Journal. Dr. B. has promised for a future number, the report of some cases of Typhoid fever, (as they are regarded by him,) which have fallen under his own observation in this vicinity. In so far as the writer's remarks are in commendation of the communication which he has briefly analysed, we trust not to incur the charge of indelicacy, from our relations to the communication and to this journal. Our readers are, of course, as well as ourselves, at liberty to make due allowance for the partiality of personal friendship.

Isolated febrile epidemics are of great importance and interest, as contributing to shed light on mooted points concerning type, contagion, etiology, &c. The facts which we were able to gather respecting the epidemic at North Boston, appear to us to establish certain conclusions as to the character, source, and diffusion of the disease, in that instance, which, if correct, have decided bearing on some of the points just enumerated; and as we venture to hope, may possess some influence in the determination of opinions on questions of importance not yet settled.—Editor.

*Account of an Epidemic Fever which occurred at North Boston, Erie County N. Y. during the months of October and November* 1843. *By Austin Flint, M. D. of Buffalo N. Y.*—Such is the title of a highly interesting article of fourteen pages in the last (July) number of the American Journal of the Medical Sciences, which possesses peculiar interest for our readers, as detailing the history of an epidemic which occurred in our midst, extremely fatal in its character, and entirely new to the physicians called upon to treat it. As none of us know at what moment another epidemic more or less similar to the one noticed may occur, and as many of our readers, probably, do not receive the valuable journal in which Dr. Flint has published his statement, it is thought that a short abstract of its principal features will prove interesting and acceptable.

It appears, then, that in September, 1843, a young man from Massachusetts stopped at Fuller's tavern in North Boston, having been unwell for several days; a diarrhoea being among the most prominent and early of his symptoms. He grew worse continually; had the same character of symptoms as the other patients hereafter to be noticed; and when first seen by a physician (which was only six days before his death) was in a typhoid condition, with diarrhoea, (or malignant dysentery as it was called) low, muttering, delirium, sordes, &c. Altogether he lived twenty-eight days after arriving at Boston. From this case, as it is very reasonably supposed, the fever spread, at about the same time, to the members of Mr. Fuller's family, and to those living nearest to his house. The first one was attacked twenty-three days after the arrival of the stranger, and consequently, five days before his death; seven of Mr.

Fuller's family had the fever of whom three died; twenty-one other cases occurred in five families, all being within ten rods of Mr. F. of whom seven died; making in all (excluding the stranger) twenty-eight cases and ten deaths. The whole population of the settlement amounted to forty-three persons only; none were attacked who were over twenty-three years old; the youngest was only one year old; a large proportion were children. Dr. Flint visited them but once, and was able to devote only about eight hours to personal observations. During this time he made an autopsical examination of the body of a child, about twelve years old, who had died with the disease; and took notes at the bed side of the history of nine cases, with the symptoms presented at the time of his visit. The late Dr. Camp afterwards wrote out a brief history of ten cases, which he placed at Dr. Flint's disposal.

From these notes we learn that after a number of days, varying from three or four to ten days in different cases, of general malaise, with more or less of lassitude and prostration of strength, pains in the head and back, anorexia, at times nausea and vomiting, with some diarrhoea, occasionally with chilly sensations and feverish symptoms, they were "taken down," in other words obliged to go to bed. Then came chills and fever, which in some cases partook of a marked remittent character, but it is not stated whether regularly so; a frequent pulse, pain in the head more or less violent; with delirium, stupor, muttering when asleep; subsultus; floccilatio; tongue generally furred, and in some cases dry with sordes on the teeth; diarrhoea and tympanites either at the time of the attack, or coming on soon afterwards; cough and expectoration in a majority of the cases; more or less restlessness and the usual concomitants of a low form of fever. These symptoms, as might be expected, did not all appear in every case, but there were always enough of them present to establish the perfect identity of the disease. Those that recovered remained in this state from three to four weeks, and then the fever left them seemingly having spent its force.

No very reliable observations were made for the characteristic eruption of typhoid fever. In several cases Dr. Baker noticed some small elevated spots about half the size of flea bites scattered over the abdomen and chest. His attention was only incidentally directed to them.

In the single post-mortem examination, there was found slight pneumonic consolidation of both lungs; the brain healthy except a slight opacity of the arachnoid, and the collection of about an ounce of serum. There were fifteen oval ulcerations in the lower third of the ileum; the glands of Peyer above those ulcerated, were thickened; the mesenteric glands corresponding to the ulcerations were enlarged and reddened; the condition of the spleen was not noted at the time, and not afterward recollected.

The particular symptoms, then, which distinguished this epidemic, were, 1st, the gradual accession of the fever; 2d the irregular chills and perspirations in many cases; 3d the headache soon relapsing into more or less delirium, stupor, and muttering; 4th the diarrhoea and tympanites present in such a large majority of the cases from the very commencement; 5th cough; 6th the duration of the fever; 7th the age of those attacked, none over twenty-three years of age; 8th the existence in one case, at least, of the lesions found in cases of death from typhoid fever, except the enlarged and softened condition of the spleen, which might or might not have been present.

These facts, in addition to the want of any reasons for considering the fever as an anomalous form of remittent fever, fully warrant Dr. Flint in the following conclusion, viz: "these considerations collectively appear to my mind to establish by irrefragable evidence, the correctness of the opinion, that the disease in question was *not* our common remittent or bilious fever, but *was* the typhoid fever of New England."

If then we are right in these opinions, we have had among us within two years an epidemic typhoid fever accompanied with an awful mortality. That it exists also among us in a sporadic form we have as little doubt, having within the last sixteen months seen seven or eight undoubted cases of the fever. We think that great credit should be awarded to Dr. F. for his article on this subject, as well as for the care with which he has drawn it up. We hope it may stimulate other physicians to note the cases of fever coming under their observation, and to give the results in this or some other Medical Journal for the common benefit of us all.

We are most lamentably deficient, all through this state, in a knowledge of the fevers which prevail in any particular locality. The only articles on this subject we can at present call to mind, (besides this of Dr. Flint's) not emanating from the physicians of New-York city, are, two published in the transactions of our state Medical Society; one by Dr. Sumner Ely, on inflammatory fever, in Vol. 5, part 1; and the other by Dr. Wm. Taylor, in Vol. 5, part 5, on typhoid fever. Indeed, in New-York city, with as abundant means as they there have, no one has as yet taken up the subject with the zeal, care, and precision of Dr. Jackson of Boston, and Drs. Gerhard and Pennock, or Dr. Stewardson of Philadelphia.

This is not as it should be, as it is probably the fact, that correct ideas of the pathology of fever do not generally prevail among the majority of the physicians of this state. Almost all of them have been taught, and still follow the popular and generally received nosology of Cullen, who classifies continued fevers into synocha, synochus, and typhus. This classification has now, by farther observations, and a more thorough knowledge of our fevers, been pretty well demonstrated to be incorrect; and if not proved absolutely to the minds of some, there is enough to lead every prudent practitioner alive to the progress of medicine during the last thirty years, to doubt its entire correctness and infallibility. On this point it behooves our profession to look upon the progress already made towards a better understanding of our fevers, and to watch the changes which time, the progress of the clearing up and settlement of our country, have made upon their character and treatment; to observe what new ones, either by contagion as in the present instance, or by some epidemic influence, have been introduced, and what old ones (if any) have ceased to exist.

We do not know as it would be worth while to give any thing of a detailed description of a case of typhoid fever, for it would be difficult to give one which would be generally applicable to particular cases. There are no one, two, or three symptoms invariably present in all cases of typhoid fever; no one, two, or three which may not be absent. A case may vary from a general sense of ailing merely, when a person knows he is unwell, and is confined the most of his time to his bed, yet cannot say what is the matter with himself, nor where he is most sick, down to the worst case of prostration, loss of intelligence, delirium, or stupor, general ataxic symptoms, with involuntary discharges, intestinal hemorrhage and death. All the varieties between these two extremes may exist, and how can one general description be given, which will comprehend all cases!

The *diagnosis* of this fever, then, often exercises peculiarly the judgment and tact of the physician. Some days may pass occasionally before he can be positive in his opinion, especially in some of the latent forms of the fever, or where the brunt of the disease is spent upon some one organ. But experience and caution will in these cases prevent the physician doing any harm by the too active use of remedies. We must remember that it is by a combination of symptoms that we are to judge of its existence. There are the slow mode of commencement, remembering all the while that its accession may be as sudden as that of any of the phlegmasiæ; the headache; vertigo; noise in the ears; deafness; delirium, at night especially; epistaxis, injection of the eyes; a peculiar dull, dirty tinge of the complexion; diarrhoea; tympanites more or less marked; some griping in the bowels; tenderness of the right iliac region; a cough coming on during the second week with sonorous and sibilant rhonchi in the lungs, indicating a bronchitis, and which may pass into a pneumonia, or a pleuro-pneumonia if the case have a malignant type; diminution of the discharge of urine from retention, or the other extreme, incontinence, with involuntary discharges from the bowels; irregular chills and fever and sweating; or but little chilliness with a dry hot skin; well marked emaciation if the case be protracted; and finally, a peculiar red papular eruption on the abdomen, and, perhaps, on the chest and limbs also; it is from a combination of a greater or lesser number of these symptoms, each coming on in its own order and time, that we are to make our diagnosis.

As a case is seen at different times and in different stages, it is liable to be mistaken for some disease of the brain; or a pneumonia; or a bad form of diarrhœa; or a sub-acute gastritis; or gastro-enteritis; or for some disease of the bladder or kidneys; or for a low remittent fever; or, finally, for the true *typhus fever*, a fever as distinct from what we are describing as typhoid fever, as scarlatina from measles. The opposite error may also be committed of mistaking these local diseases for typhoid fever. The *typhoid condition*, or state of the system, ought always to be carefully distinguished from *typhoid fever*.

The *prognosis* of typhoid fever is always grave. It is a very serious disease to be afflicted with. The mortality varies greatly, however, in different epidemics; from over a third, as in the epidemic just noticed, to only one in twenty, as in the epidemic at Manlius, described by Dr. Taylor in the address already referred to. In simple sporadic cases, uninfluenced by epidemic causes, the mortality varies from one death in six of those attacked, to one in ten.

Cases complicated with irregular but severe chills, we have found more fatal than where these did not exist; profuse and continued sweating, constant ataxic symptoms, maniacal delirium or great stupor, retention of urine requiring the daily use of the catheter, involuntary discharges, intestinal hemorrhage, are symptoms betokening danger, while the occurrence of pneumonia, or peritonitis, renders recovery exceedingly doubtful.

We have, besides, one constant source of apprehension in this fever. We never know, nor have we the means of knowing, the extent of the intestinal ulcerations. All may seem to be progressing well, the pulse, the skin, the tongue, the intelligence, the discharges from the bowels may all be in as good a state as we could wish, yet there may be one small ulcer working its silent and unseen way toward perforation until its work is accomplished, and in a few hours, after the most agonizing sufferings, our patient may be a corpse! Our prognosis ought always to be guarded, if we mean to speak and practice upon strictly rational and scientific principles.

Having already extended this notice to an unreasonable length, our remarks upon the treatment must necessarily be brief. A great many experiments have been made in the French Hospitals, as to the best mode of treatment, and they seem to have settled down upon the purgative plan. We confess we doubt the propriety of any thing like purging, except, perhaps, for the first few days, when it may seem to be especially demanded. Our treatment must be adapted to circumstances according to the different degrees of severity of the fever. It must be rationally eclectic, having a prudent regard to the future; bear in mind that the fever *must run its course*, and that our great object should be to conduct it to a safe termination. Cases will occur where but little treatment will be needed beyond good nursing; and other cases will be met with, which will put to the test, all the prudence, skill, and resources, of the Physician. Let us all bear one principle in mind, *to do no harm* by our remedies and medicines. An improper bleeding, or emetic, or a dose of physic, even, ill-timed, may cost our patient his life. Let us, therefore, keep constantly before us a just sense of our responsibility. G. N. B.

*Buffalo, August*, 1845.

